# The genome sequence of the common green Tenthredo, *Tenthredo mesomela* (Linnaeus, 1758)

**DOI:** 10.12688/wellcomeopenres.18992.1

**Published:** 2023-02-15

**Authors:** Steven Falk, University of Oxford and Wytham Woods Genome Acquisition Lab, Darwin Tree of Life Barcoding collective, Wellcome Sanger Institute Tree of Life programme, Wellcome Sanger Institute Scientific Operations: DNA Pipelines collective, Tree of Life Core Informatics collective, Andrew Green, Gavin R Broad, Darwin Tree of Life Consortium

**Affiliations:** 1 Independent Researcher, Kenilworth, Warwickshire, UK; 2 Sawfly Recording Scheme, Bedford, Bedfordshire, UK; 3 Natural History Museum, London, UK

**Keywords:** Tenthredo mesomela, common green-Tenthredo, genome sequence, chromosomal, Hymenoptera

## Abstract

We present a genome assembly from an individual female *Tenthredo mesomela* (the common green Tenthredo; Arthropoda; Insecta; Hymenoptera; Tenthredinidae). The genome sequence is 393 megabases in span. Most of the assembly is scaffolded into 10 chromosomal pseudomolecules. The mitochondrial genome has also been assembled and is 15.6 kilobases in length. Gene annotation of this assembly on Ensembl has identified 11,086 protein coding genes.

## Species taxonomy

Eukaryota; Metazoa; Ecdysozoa; Arthropoda; Hexapoda; Insecta; Pterygota; Neoptera; Endopterygota; Hymenoptera; Tenthredinoidea; Tenthredinidae; Tenthredininae; *Tenthredo*; *Tenthredo mesomela* (Linnaeus, 1758) (NCBI:txid222778).

## Background

The *Tenthredo* genus is large with over one thousand species distributed across the holarctic region. There are 30 species present in Britain. Within this genus, numerous species groups and subspecies have been identified. *Tenthredo mesomela* falls within the subgenus *Eurogaster*, together in Britain with *Tenthredo obsoleta* and *Tenthredo mioceras*. *Tenthredo obsoleta* and *mioceras* are both associated with more upland areas, whilst mesomela is widespread across lowland and upland areas ( Benson, 1943; Benson, 1952).

*Tenthredo mesomela* is a large (9.5 to 13 mm) green sawfly marked with black on the head, antennae, thorax, abdomen, and legs. It is distributed across Europe and Asia. Adults can be distinguished from the similar *mioceras* by a combination of green mesopleura with a black sub-vertical stripe, and dark hairs on the head. The adults are highly predatory and play a crucial role in controlling pest species. Prey species are known to include Diptera such as *Calliphora*, Scatophaga, and Bibionid flies. The larvae are polyphagous, feeding on herbaceous and woody plants from a broad range of plant families ( Liston, 1995), but are not considered to be a pest of agricultural or horticultural significance. The species is univoltine, with adults on the wing from May to July.

Barcode Index Number (BIN) divergence is present in *T. mesomela* barcoding ( Schmidt *et al.*, 2017). The specimen studied here, from Oxfordshire, England, falls in a larger cluster of barcodes (BOLD:AAD3086), which contains specimens from all over Western, Northern and Central Europe. A second smaller BIN (BOLD:ABZ0292) for *mesomela* is represented by fewer specimens, from Scandinavia and Russia (Broad, G., pers comms). To date, very few Hymenoptera genomes have been sequenced, and this is especially true for the sawflies. The *T. mesomela* genome will help our understanding of the evolution of this group. This is only the second genome of a *Tenthredo* species, the other being *Tenthredo notha* ( Falk & Broad, 2022). Knowledge of sawfly evolution will benefit from the comparative analysis of genomes from closely and distantly related species.

### Genome sequence report

**Figure 1.**
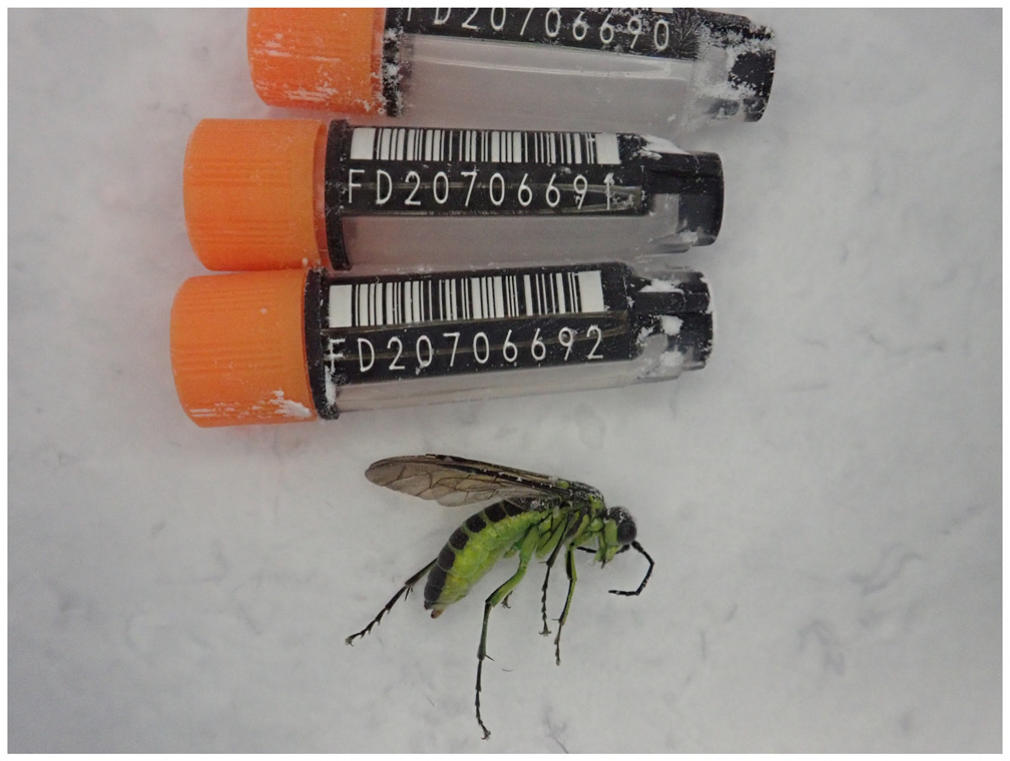
Photograph of the *Tenthredo mesomela* (iyTenMeso1) specimen used for genome sequencing.

The genome was sequenced from one female *Tenthredo mesomela* ( Figure 1) collected from Wytham Woods, Oxfordshire (latitude 51.76, longitude –1.33). A total of 60-fold coverage in Pacific Biosciences single-molecule HiFi long reads was generated. Primary assembly contigs were scaffolded with chromosome conformation Hi-C data. Manual assembly curation corrected 85 missing or mis-joins and removed 19 haplotypic duplications, reducing the assembly length by 1.57% and the scaffold number by 33.66%, and increasing the scaffold N50 by 52.64%.

**Figure 2.**
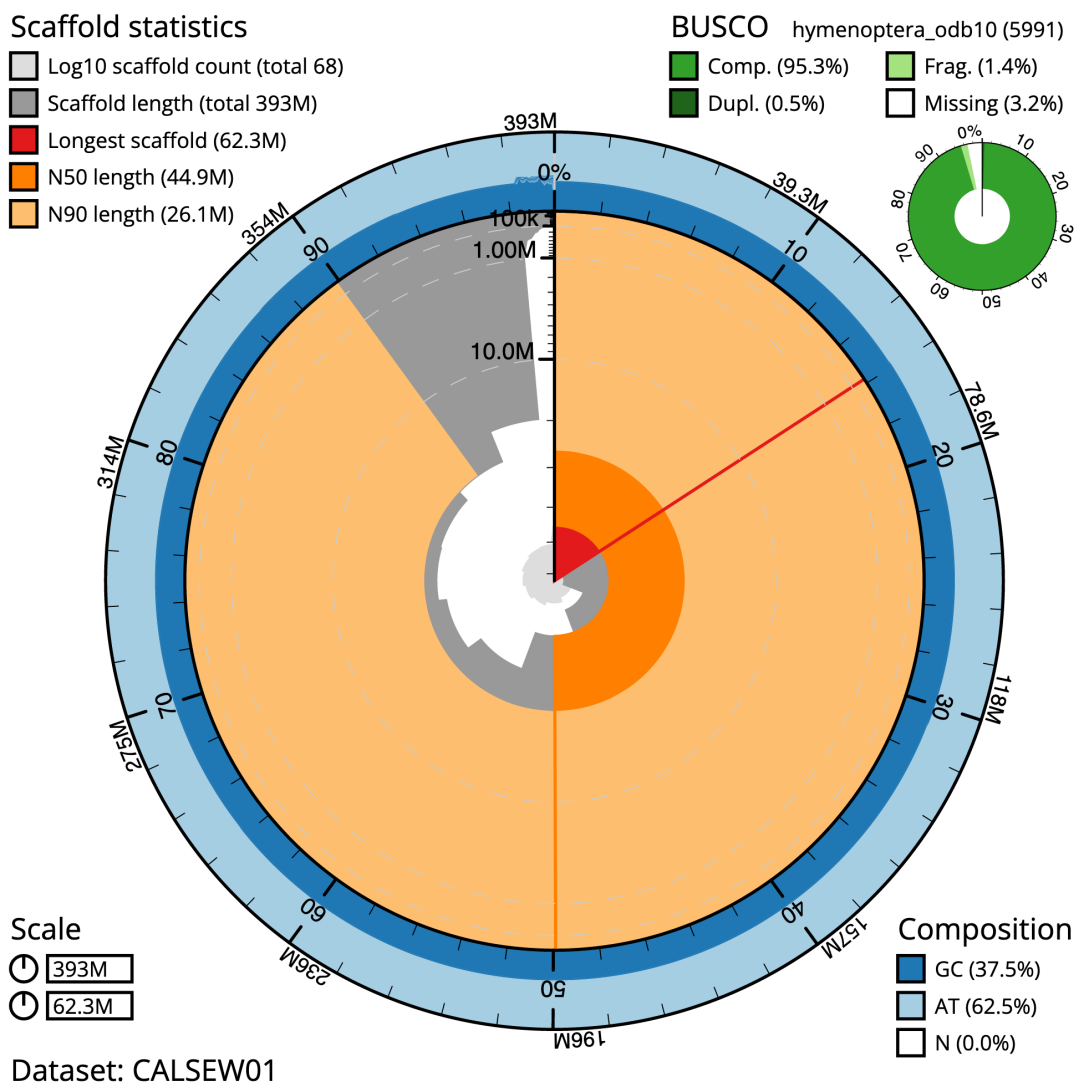
Genome assembly of *Tenthredo mesomela*, iyTenMeso1.1: metrics. The BlobToolKit Snailplot shows N50 metrics and BUSCO gene completeness. The main plot is divided into 1,000 size-ordered bins around the circumference with each bin representing 0.1% of the 392,852,752 bp assembly. The distribution of scaffold lengths is shown in dark grey with the plot radius scaled to the longest sequence present in the assembly (62,304,763 bp, shown in red). Orange and pale-orange arcs show the N50 and N90 scaffold lengths (44,941,172 and 26,137,029 bp), respectively. The pale grey spiral shows the cumulative scaffold count on a log scale with white scale lines showing successive orders of magnitude. The blue and pale-blue area around the outside of the plot shows the distribution of GC, AT and N percentages in the same bins as the inner plot. A summary of complete, fragmented, duplicated and missing BUSCO genes in the hymenoptera_odb10 set is shown in the top right. An interactive version of this figure is available at https://blobtoolkit.genomehubs.org/view/iyTenMeso1.1/dataset/CALSEW01/snail.

The final assembly has a total length of 392.8 Mb in 67 sequence scaffolds with a scaffold N50 of 44.9 Mb ( Table 1). Most (98.46%) of the assembly sequence was assigned to 10 chromosomal-level scaffolds. Chromosome-scale scaffolds confirmed by the Hi-C data are named in order of size ( Figure 2– Figure 5; Table 2). The individual is diploid and hence female. While not fully phased, the assembly deposited is of one haplotype. Contigs corresponding to the second haplotype have also been deposited. The assembly has a BUSCO v5.3.2 ( Manni *et al.*, 2021) completeness of 95.3% (single 94.9%, duplicated 0.5%) using the hymenoptera_odb10 reference set ( *n* = 5,911).

**Table 1.** Genome data for *Tenthredo mesomela,* iyTenMeso1.1.

Project accession data		
Assembly identifier	iyTenMeso1.1	
Species	*Tenthredo mesomela*	
Specimen	iyTenMeso1	
NCBI taxonomy ID	222778	
BioProject	PRJEB52925	
BioSample ID	SAMEA10167061	
Isolate information		
Assembly metrics *		*Benchmark*
Consensus quality (QV)	62.7	*≥ 50*
*k*-mer completeness	100%	*≥ 95%*
BUSCO **	C:95.3%[S:94.9%,D:0.5%], F:1.4%,M:3.2%,n:5,991	*C ≥ 95%*
Percentage of assembly mapped to chromosomes	98.46%	*≥ 95%*
Sex chromosomes	N/A	*localised homologous pairs*
Organelles	Mitochondrial genome assembled	*complete single alleles*
Raw data accessions		
PacificBiosciences SEQUEL II	ERR9793198	
Hi-C Illumina	ERR9767809	
PolyA RNA-Seq Illumina	ERR10123703	
Genome assembly		
Assembly accession	GCA_943736025.1	
*Accession of alternate haplotype*	GCA_943736035.1	
Span (Mb)	392.8	
Number of contigs	245	
Contig N50 length (Mb)	3.4	
Number of scaffolds	67	
Scaffold N50 length (Mb)	44.9	
Longest scaffold (Mb)	62.3	
Genome annotation		
Number of protein-coding genes	11,086	
Number of non-coding genes	1,869	
Number of gene transcripts	18,839	

* Assembly metric benchmarks are adapted from column VGP-2020 of “Table 1: Proposed standards and metrics for defining genome assembly quality” from ( Rhie *et al.*, 2021). ** BUSCO scores based on the hymenoptera_odb10 BUSCO set using v5.3.2. C = complete [S = single copy, D = duplicated], F = fragmented, M = missing, n = number of orthologues in comparison. A full set of BUSCO scores is available at https://blobtoolkit.genomehubs.org/view/iyTenMeso1.1/dataset/CALSEW01/busco.

**Figure 3.**
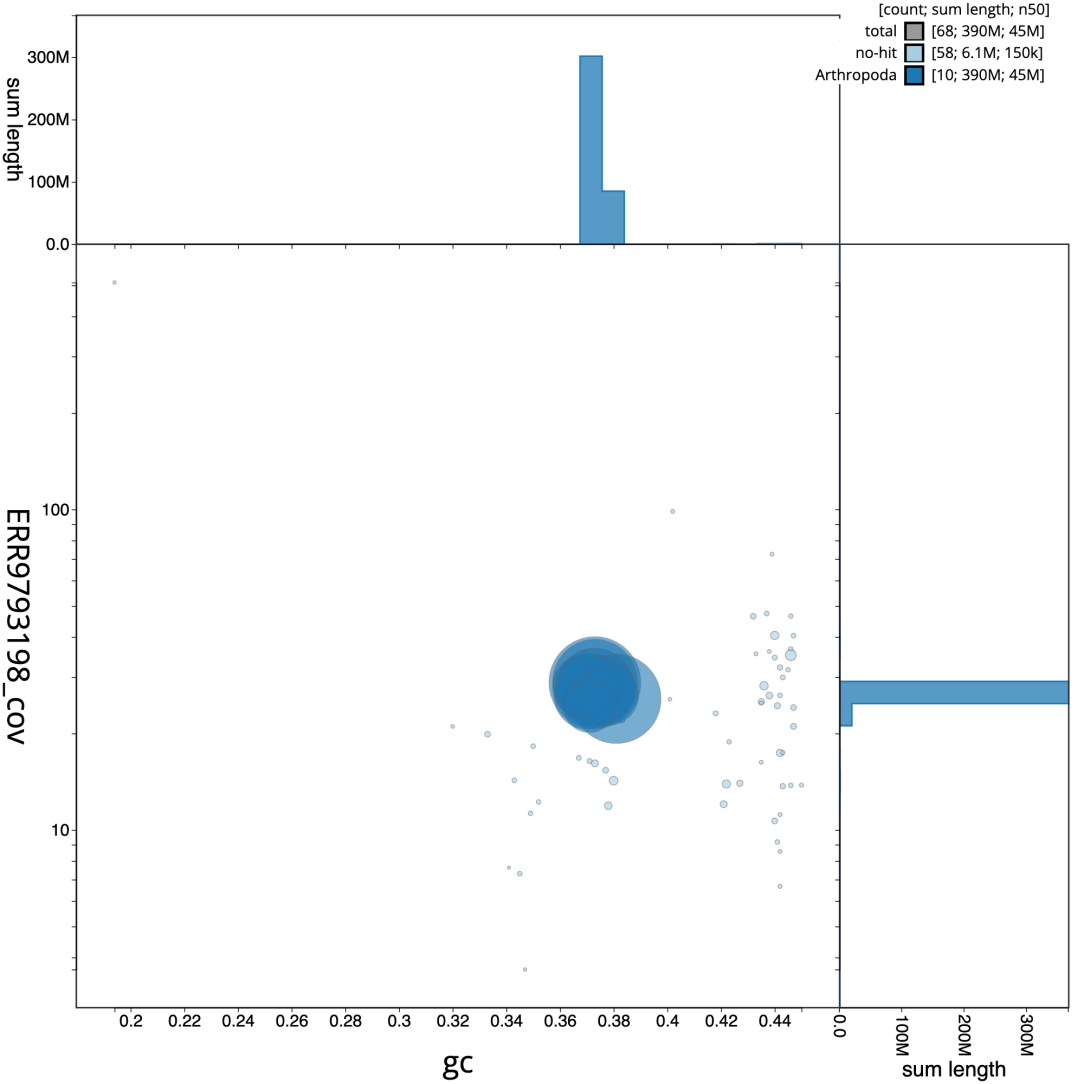
Genome assembly of *Tenthredo mesomela*, iyTenMeso1.1: GC coverage. BlobToolKit GC-coverage plot. Scaffolds are coloured by phylum. Circles are sized in proportion to scaffold length. Histograms show the distribution of scaffold length sum along each axis. An interactive version of this figure is available at https://blobtoolkit.genomehubs.org/view/iyTenMeso1.1/dataset/CALSEW01/blob.

**Figure 4.**
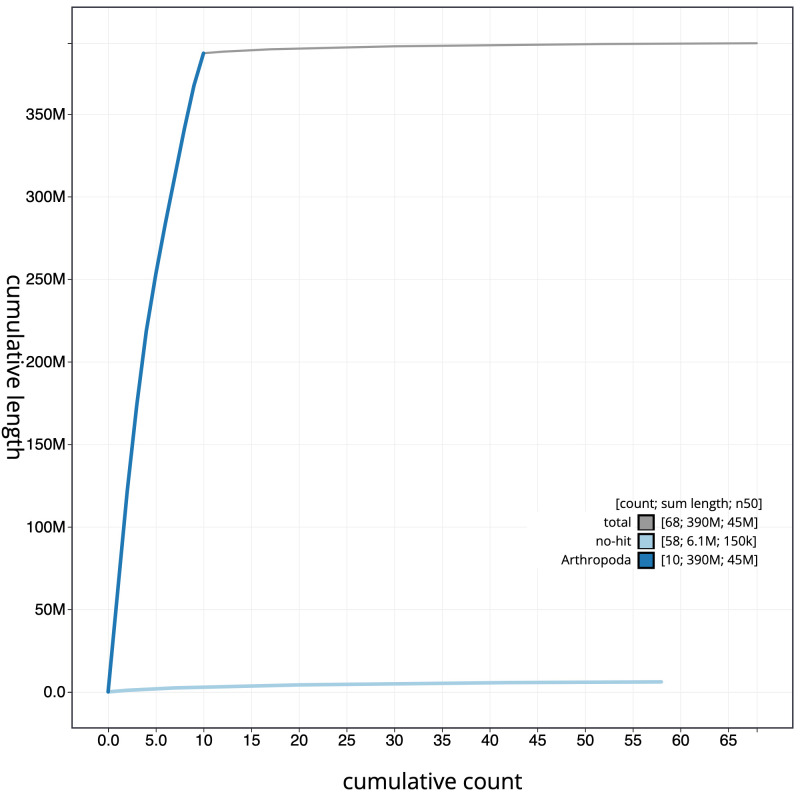
Genome assembly of *Tenthredo mesomela*, iyTenMeso1.1: cumulative sequence. BlobToolKit cumulative sequence plot. The grey line shows cumulative length for all scaffolds. Coloured lines show cumulative lengths of scaffolds assigned to each phylum using the buscogenes taxrule. An interactive version of this figure is available at https://blobtoolkit.genomehubs.org/view/iyTenMeso1.1/dataset/CALSEW01/cumulative.

**Figure 5.**
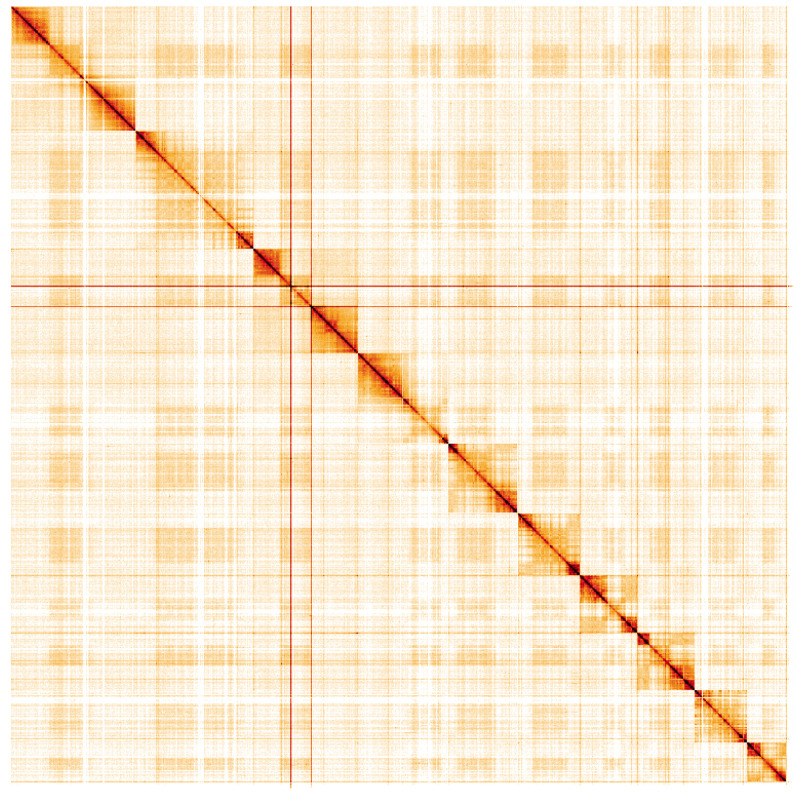
Genome assembly of *Tenthredo mesomela*, iyTenMeso1.1: Hi-C contact map. Hi-C contact map of the iyTenMeso1.1 assembly, visualised using HiGlass. Chromosomes are shown in order of size from left to right and top to bottom. An interactive version of this figure may be viewed at https://genome-note-higlass.tol.sanger.ac.uk/l/?d=MsYCnkSNRjCIRBBaafQhWQ.

**Table 2.** Chromosomal pseudomolecules in the genome assembly of *Tenthredo mesomela*, iyTenMeso1.

INSDC accession	Chromosome	Size (Mb)	GC%
OX031013.1	1	62.3	37.3
OX031014.1	2	59.02	38.1
OX031015.1	3	52.12	37.3
OX031016.1	4	44.94	37.3
OX031017.1	5	34.58	37.5
OX031018.1	6	30.72	37
OX031019.1	7	28.84	36.9
OX031020.1	8	28.58	37.1
OX031021.1	9	26.14	37.8
OX031022.1	10	19.55	37.1
OX031023.1	MT	0.02	19.5

### Genome annotation report

The GCA_943736025.1 genome assembly was annotated using the Ensembl rapid annotation pipeline ( Table 1; https://rapid.ensembl.org/Tenthredo_mesomela_GCA_943736025.1/). The resulting annotation includes 18,839 transcribed mRNAs from 11,086 protein-coding and 1,869 non-coding genes.

## Methods

### Sample acquisition and nucleic acid extraction

Two *T. mesomela* specimens (iyTenMeso1: female; iyTenMeso2: male) were collected by netting in Wytham Woods, Oxfordshire (biological vice-county: Berkshire), UK (latitude 51.76, longitude –1.33) on 31 May 2021. The specimens were collected and identified by Steven Falk (independent researcher) and then snap-frozen on dry ice.

DNA was extracted at the Tree of Life laboratory, Wellcome Sanger Institute (WSI). The iyTenMeso1 sample was weighed and dissected on dry ice with tissue set aside for Hi-C sequencing. Thorax tissue was disrupted using a Nippi Powermasher fitted with a BioMasher pestle. High molecular weight (HMW) DNA was extracted using the Qiagen MagAttract HMW DNA extraction kit. HMW DNA was sheared into an average fragment size of 12–20 kb in a Megaruptor 3 system with speed setting 30. Sheared DNA was purified by solid-phase reversible immobilisation using AMPure PB beads with a 1.8X ratio of beads to sample to remove the shorter fragments and concentrate the DNA sample. The concentration of the sheared and purified DNA was assessed using a Nanodrop spectrophotometer and Qubit Fluorometer and Qubit dsDNA High Sensitivity Assay kit. Fragment size distribution was evaluated by running the sample on the FemtoPulse system.

RNA was extracted from abdomen tissue of iyTenMeso2 in the Tree of Life Laboratory at the WSI using TRIzol, according to the manufacturer’s instructions. RNA was eluted in 50 μl RNAse-free water and its concentration assessed using a Nanodrop spectrophotometer and Qubit Fluorometer using the Qubit RNA Broad-Range (BR) Assay kit. Analysis of the integrity of the RNA was done using Agilent RNA 6000 Pico Kit and Eukaryotic Total RNA assay.

### Sequencing

Pacific Biosciences HiFi circular consensus and 10X Genomics read cloud DNA sequencing libraries were constructed according to the manufacturers’ instructions. Poly(A) RNA-Seq libraries were constructed using the NEB Ultra II RNA Library Prep kit. DNA and RNA sequencing were performed by the Scientific Operations core at the WSI on Pacific Biosciences SEQUEL II (HiFi) and Illumina NovaSeq 6000 instruments. Hi-C data were also generated from head tissue of iyTenMeso2 using the Arima v2kit and sequenced on the Illumina NovaSeq 6000 instrument.

### Genome assembly

Assembly was carried out with Hifiasm ( Cheng *et al.*, 2021) and haplotypic duplication was identified and removed with purge_dups ( Guan *et al.*, 2020). The assembly was then scaffolded with Hi-C data ( Rao *et al.*, 2014) using YaHS ( Zhou *et al*, 2023). The assembly was checked for contamination and corrected as described previously ( Howe *et al.*, 2021). Manual curation was performed using HiGlass ( Kerpedjiev *et al.*, 2018) and Pretext ( Harry, 2022). The mitochondrial genome was assembled using MitoHiFi ( Uliano-Silva *et al.*, 2022), which performed annotation using MitoFinder ( Allio *et al.*, 2020). The genome was analysed and BUSCO scores generated within the BlobToolKit environment ( Challis *et al.*, 2020). Table 3 contains a list of all software tool versions used, where appropriate.

**Table 3.** Software tools and versions used.

Software tool	Version	Source
BlobToolKit	3.5.2	Challis *et al.*, 2020
Hifiasm	0.16.1-r375	Cheng *et al.*, 2021
HiGlass	1.11.6	Kerpedjiev *et al.*, 2018
MitoHiFi	2	Uliano-Silva *et al.*, 2022
PretextView	0.2	Harry, 2022
purge_dups	1.2.3	Guan *et al.*, 2020
YaHS	yahs-1.1.91eebc2	Zhou *et al.*, 2023

### Genome annotation

The Ensembl gene annotation system ( Aken *et al.*, 2016) was used to generate annotation for the *T. mesomela* assembly (GCA_943736025.1). Annotation was created primarily through alignment of transcriptomic data to the genome, with gap filling via protein to-genome alignments of a select set of proteins from UniProt ( UniProt Consortium, 2019).

### Ethics and compliance issues

The materials that have contributed to this genome note have been supplied by a Darwin Tree of Life Partner. The submission of materials by a Darwin Tree of Life Partner is subject to the Darwin Tree of Life Project Sampling Code of Practice. By agreeing with and signing up to the Sampling Code of Practice, the Darwin Tree of Life Partner agrees they will meet the legal and ethical requirements and standards set out within this document in respect of all samples acquired for, and supplied to, the Darwin Tree of Life Project. All efforts are undertaken to minimise the suffering of animals used for sequencing. Each transfer of samples is further undertaken according to a Research Collaboration Agreement or Material Transfer Agreement entered into by the Darwin Tree of Life Partner, Genome Research Limited (operating as the Wellcome Sanger Institute), and in some circumstances other Darwin Tree of Life collaborators.

## Data Availability

European Nucleotide Archive: *Tenthredo mesomela* (common green-Tenthredo). Accession number PRJEB52925; https://identifiers.org/ena.embl/PRJEB52925. ( Wellcome Sanger, 2022) The genome sequence is released openly for reuse. The *Tenthredo mesomela* genome sequencing initiative is part of the Darwin Tree of Life (DToL) project. All raw sequence data and the assembly have been deposited in INSDC databases. Raw data and assembly accession identifiers are reported in Table 1. Members of the University of Oxford and Wytham Woods Genome Acquisition Lab are listed here: https://doi.org/10.5281/zenodo.4789928. Members of the Darwin Tree of Life Barcoding collective are listed here: https://doi.org/10.5281/zenodo.4893703. Members of the Wellcome Sanger Institute Tree of Life programme are listed here: https://doi.org/10.5281/zenodo.4783585. Members of Wellcome Sanger Institute Scientific Operations: DNA Pipelines collective are listed here: https://doi.org/10.5281/zenodo.4790455. Members of the Tree of Life Core Informatics collective are listed here: https://doi.org/10.5281/zenodo.5013541. Members of the Darwin Tree of Life Consortium are listed here: https://doi.org/10.5281/zenodo.4783558.
