# Supplementary material for: The genome sequence of the common green Tenthredo, Tenthredo mesomela Linnaeus, 1758
Source: Wellcome Open Res. 2024 Oct 11;8:80. Originally published 2023 Feb 15. [Version 2] doi: 10.12688/wellcomeopenres.18992.2 (PMC11525098; doi:10.12688/wellcomeopenres.18992.2)
Supplement: Peer review history

# Reviewer report: Reviewer response for version 2

**DOI:** 10.21956/wellcomeopenres.25598.r107444

**Published:** 2024-11-15

**Authors:** Marcus C Stensmyr

**Affiliations:** 1 Lund University, Lund, Sweden

The manuscript details the genome sequencing, assembly and annotation of the common green sawfly ( *Tenthredo mesomela). *The manuscript has been revised, and now encompass more details with regards to the sequencing technology employed. I see no need for further revisions. The genome is part of the TOL project, and has been sequenced, assembled and annotated using their state-of-the-art pipeline.

Are sufficient details of methods and materials provided to allow replication by others?

Yes

Is the rationale for creating the dataset(s) clearly described?

Yes

Are the datasets clearly presented in a useable and accessible format?

Yes

Are the protocols appropriate and is the work technically sound?

Yes

Reviewer Expertise:

Drosophila genomics and neurogenetics.

I confirm that I have read this submission and believe that I have an appropriate level of expertise to confirm that it is of an acceptable scientific standard.

# Reviewer report: Reviewer response for version 2

**DOI:** 10.21956/wellcomeopenres.25598.r107448

**Published:** 2024-10-30

**Authors:** Ruiqi Li

**Affiliations:** 1 University of Colorado Boulder, Boulder, Colorado, USA

Falk et al. presented a high quality genome of the common green Tenthredo. I think the revised version has addressed all the previous comments. I only have a few minor comments.

 1. It’s great the last paragraph of introduction mentioned the significance of sequencing this genome, but it would be great if the authors could specify which specific questions could be answered using comparative genomics. See some examples in Li et al 2024.

 2. Adding a scale bar to figure 1?

Are sufficient details of methods and materials provided to allow replication by others?

Yes

Is the rationale for creating the dataset(s) clearly described?

Yes

Are the datasets clearly presented in a useable and accessible format?

Yes

Are the protocols appropriate and is the work technically sound?

Yes

Reviewer Expertise:

Genomics

I confirm that I have read this submission and believe that I have an appropriate level of expertise to confirm that it is of an acceptable scientific standard.

## References

[1] : The genome sequence of the giant clam, Tridacna gigas (Linnaeus, 1758). *Wellcome Open Res* .2024;9: 10.12688/wellcomeopenres.21136.1 145 10.12688/wellcomeopenres.21136.1 38800516 PMC11116938

[2] : Comparative transcriptomics revealed parallel evolution and innovation of photosymbiosis molecular mechanisms in a marine bivalve. *Proc Biol Sci* .2024;291(2023) : 10.1098/rspb.2023.2408 20232408 10.1098/rspb.2023.2408 38807516 PMC11285757

# Reviewer report: Reviewer response for version 1

**DOI:** 10.21956/wellcomeopenres.21056.r58044

**Published:** 2023-06-30

**Authors:** Bao-Zhen Hua

**Affiliations:** 1 Northwest A&F University, Shaanxi, China

The manuscript entitled “The genome sequence of the common green Tenthredo, Tenthredo mesomela (Linnaeus, 1758)”, presents the genome sequence of the second species of sawflies. Generally, the manuscript is well-written, and contributes new knowledge of genomics of Hymenoptera, thus deserves to be published in this journal. I recommend it to be indexed after a minor revision.

1. Page 3, Background, Passage 1, Line 2: Please change “the holarctic region” to “the Holarctic Region”;
2. Page 3, Background, Passage 1, Line 8: “mesomela” is the specific name of a species, thus it should be in italics;
3. Page 3, Background, Passage 2, Line 8: “Scatophaga” is a scientific name of a genus, so it should be in italics.

Are sufficient details of methods and materials provided to allow replication by others?

Yes

Is the rationale for creating the dataset(s) clearly described?

Yes

Are the datasets clearly presented in a useable and accessible format?

Yes

Are the protocols appropriate and is the work technically sound?

Yes

Reviewer Expertise:

systematist and phylogenetist.

I confirm that I have read this submission and believe that I have an appropriate level of expertise to confirm that it is of an acceptable scientific standard.

# Reviewer report: Reviewer response for version 1

**DOI:** 10.21956/wellcomeopenres.21056.r54993

**Published:** 2023-03-07

**Authors:** Craig Michell

**Affiliations:** 1 Department of Environmental and Biological Sciences, University of Eastern Finland, Joensuu, Finland

**Summary:**

 The authors here present a high-quality genome of an individual female from the species *Tenthredo mesomela* (Linnaeus, 1758). The reported genome is 392,852,752 bases in length has a high contiguity and level of completeness. The genome will provide an excellent resource for further studies into the evolution of Hymenopterans.

**Methods:**

 The authors used appropriate methods to assemble the genome of this species. The technologies used for the genome assembly include 60X genome coverage PacBio HiFi sequencing, 10X genomics DNA cloud reads and Hi-C sequencing.

 The genome was annotated using the Ensembl gene annotation pipeline.

**Datasets:**

 The datasets presented are clear and freely accessible online.

**Comments:**

 I have mostly minor comments related to making the methods more reproducible especially for people interested in performing a similar project.

- Can you comment on the difference between your reported genome length and that reported on the rapid ensembl website linked in the genome annotation report?

I would like to see some clarification of important points in the methods:

- You mention dissecting the female and saving the tissue for HiC, yet the HiC library was made from the male. Can you clarify which specimen was used for the HiC library?
- What was your DNA input amount for the HiFi libraries, 10X genomics DNA cloud, Hi-C, Transcriptomes?
- What was the average size of the HMW DNA used for the HiFi library?
- What was the quality of the RNA, RIN >7?
- What were the 10X genomic DNA cloud read data used for? You mention creating them, and then they are never mentioned again.

Regarding the Hi-C data:

- If this was created from a male, I presume that the male is haploid in this species like many Hymenoptera, did this affect your phasing? Or was only the HiFi data used for phasing?
- How was the Hi-C data mapped to the genome? BWA mem? Bowtie? Some other program?
- How was this data processed? How did you mark duplicates in this dataset?

Annotation:

- How did you align your RNA to the genome?
- What is the select set of proteins from UniProt?

Are sufficient details of methods and materials provided to allow replication by others?

No

Is the rationale for creating the dataset(s) clearly described?

Yes

Are the datasets clearly presented in a useable and accessible format?

Yes

Are the protocols appropriate and is the work technically sound?

Yes

Reviewer Expertise:

Genome assembly of insects and other higher Eukaryotes.

I confirm that I have read this submission and believe that I have an appropriate level of expertise to confirm that it is of an acceptable scientific standard, however I have significant reservations, as outlined above.

# Response

**Published:** 2024-10-27

**Authors:** Tree of Life Team Sanger

**Affiliations:** Wellcome Trust Sanger Institute, UK

Thank you for reviewing this data note. We have responded to your comments below.

  

- Can you comment on the difference between your reported genome length and that reported on the rapid ensembl website linked in the genome annotation report?

**Response:** The assembly length is reported as 392.8 Mb in the Abstract and genome sequence report. We report the assembly length as 392,852,752 bases in Figure 2, including all assembled molecules, while Ensembl at the ENA reports 392,837,180 bases. The difference appears to be the length of the mitochondrial genome (15.6 kb).

- You mention dissecting the female and saving the tissue for HiC, yet the HiC library was made from the male. Can you clarify which specimen was used for the HiC library?

**Response:** We have now added a section to Table 1 which specifies which specimens are used for each sequencing technology. To clarify, the final Hi-C data used in the assembly was from the male specimen, iyTenMeso2.

- What was your DNA input amount for the HiFi libraries, 10X genomics DNA cloud, Hi-C, transcriptomes?
- What was the average size of the HMW DNA used for the HiFi library?
- What was the quality of the RNA, RIN >7?

**Response**: We have expanded the Methods information section on library preparation sequencing to provide more detail.

- What were the 10X genomic DNA cloud read data used for? You mention creating them, and then they are never mentioned again.

**Response:** Although 10X data was generated, we have not used it in the assembly for polishing, and so we have removed any mention of 10X data from this data note. Regarding the Hi-C data:

- If this was created from a male, I presume that the male is haploid in this species like many Hymenoptera, did this affect your phasing? Or was only the HiFi data used for phasing?
- How was the Hi-C data mapped to the genome? BWA mem? Bowtie? Some other program?
- How was this data processed? How did you mark duplicates in this dataset?

**Response:** Hi-C data was not used for phasing in this assembly. Hifiasm was used in primary mode and purge-dups was used after the original assembly of Hi-Fi reads. We have now specified that bwa-mem2 was used for Hi-C mapping. Manual curation of the assembly was performed in PretextView. Annotation:

- How did you align your RNA to the genome?
- What is the select set of proteins from UniProt?

**Response:** Annotation is performed by Ensembl at ENA, not by the Wellcome Sanger Institute, and so we have not provided details of the annotation method. The methods are outlined in the link: https://rapid.ensembl.org/info/genome/genebuild/anno.html.
